# A novel technique for managing symptomatic spinal cysts using epiduroscopic neural laser decompression: technical note and preliminary results

**DOI:** 10.1186/s13018-018-0849-3

**Published:** 2018-06-04

**Authors:** Seung-Kook Kim, Byoung-Hoi Lee, Moon-Bok Song, Su-Chan Lee

**Affiliations:** 1 Department of Neurosurgery, Spine Center, Himchan Hospital, 118 Yongdam-ro, Yunsoo-gu, Incheon, 21927 South Korea; 2 Joint and Arthritis Research, Orthopaedic Surgery, Himchan Hospital, 120 Sinmok-ro, Yangcheon-gu, Seoul, South Korea; 3 Department of Orthopedic Surgery, Himchan Hospital, 118 Yongdam-ro, Yunsoo-gu, Incheon, 21927 South Korea

**Keywords:** Spinal cysts, Symptomatic, ENLD, Endoscopic spine surgery, Laser surgery

## Abstract

**Background:**

Benign spinal cysts are relatively common, but can cause significant pain. However, consensus regarding the best method for treating these cysts has not been established. We aimed to examine the usefulness of epiduroscopic neural laser decompression (ENLD), a novel percutaneous treatment, for treating lumbo-sacral cysts.

**Methods:**

Ten patients (6 men, 4 women; mean age 45.5 years) with benign lumbo-sacral cysts underwent ENLD. The lumbo-sacral cysts were caused by multiple pathophysiologies and displayed different characteristics. Cysts were evaluated using a recorded epiduroscopic procedure video, magnetic resonance imaging (MRI), and electronic medical records. In all patients, MRI identified cysts with well-defined margins that were compressing the nerves in the lumbo-sacral region and were associated with the pain symptoms of the patients. Retrospectively, we reviewed a series of consecutive patients who underwent surgery (two with discal cysts, four with facet cysts, and four with Tarlov cysts). Low back/leg pain was evaluated using a 1–10 visual analog scale. Functional improvement was evaluated using Oswestry Disability Index scores. Outcomes were evaluated pre- and post-operatively and 1 year post-surgery.

**Results:**

Patients were examined between May 2016 and August 2017. Average pain scores improved from 4.7 pre-surgery to 1.8 post-surgery (low back; *p* < .001) and from 5.8 pre-surgery to 1.6 post-surgery (leg; *p* < .001). Disability scores decreased from 27.2% pre-surgery to 14.6% post-surgery.

**Conclusion:**

Currently, no standard treatment strategy for symptomatic spinal cysts exists. These results show that ENLD using a Holmium: YAG laser can be useful in treating symptomatic benign spinal cysts.

**Trial registration:**

Not applicable as this is a retrospective chart review.

## Background

Benign spinal cysts are a relatively common type of expanding lesion that form in the spinal canal. Benign cysts can be the cause of both chronic back pain and lower extremity radiculopathy [1]. The negative effects of benign cysts are attributed to nerve compression caused by the hydrostatic pressure of cerebrospinal fluid (CSF) or nerve irritation.

Till date, there are only been seven options for treating benign cysts—four open invasive options and three percutaneous non-invasive procedures. Open procedures for treating benign cysts include (1) laminectomy for cyst decompression and nerve root resection [2–4], (2) laminoplasty and cyst fenestration [5], (3) incision and cyst drainage with plication of cyst [6], (4) and lumbo-peritoneal CSF shunting [7]. Percutaneous procedures for addressing benign cysts include (1) computed tomography (CT)-guided cyst rupture [8, 9], (2) fluoroscopic fenestration and injections [10], and (3) trans-foraminal approach and cyst removal or fenestration [11]. However, these surgical methods have several important drawbacks, including the possibility of triggering neurological deficits, technical difficulty, and possible complications such as instability of the spine. In addition, while CT and fluoroscopic procedures are non-invasive, confirmation of cyst rupture using these techniques is not possible. The trans-foraminal approach can be applied only in a few cases without degenerative changes.

Since Choy et al. first introduced epiduroscopic neural laser decompression (ENLD), the technique and instrumentation of ENLD has significantly improved [12]. Due to the development of endoscopic cameras allowing surgical visibility and the effectiveness of laser ablation, ENLD is becoming a viable alternative for invasive operative procedures [13]. However, to the best of our knowledge, the use of ENLD to treat cystic spine lesions has not yet been reported. The purpose of this study was to introduce the ENLD technique as a novel percutaneous treatment for spinal symptomatic lesions.

## Methods

### Patients and study design

Ten adult patients participated in this prospective study between May 2016 and August 2017. We received approval from the Institutional Review Board of Himchan Hospital Health System (No. 112294-01-201801-01), and all participants provided written informed consent.

### Inclusion and exclusion criteria

Participants were eligible to participate in the study if they exhibited single-lesion, low back and/or radicular pain, with clear evidence of cystic lesions on magnetic resonance imaging (MRI), and correlated back and/or leg radiating pain which had not responded to conservative treatment (medication and physiotherapy) within 6 weeks. Participant age (in years), sex (male/female), and estimated duration of pain (in months) were collected during epiduroscopic video recording. Exclusion criteria for the study included (1) evidence of intradural mass lesion or suggestive malignant lesions, (2) presence of multiple cystic lesions or metastatic lesions on cross-sectional MR images, and (3) pain due to infection, instability, herniated lumbar disc, and spondylolisthesis.

### Surgical procedure

**Fig. 1:**
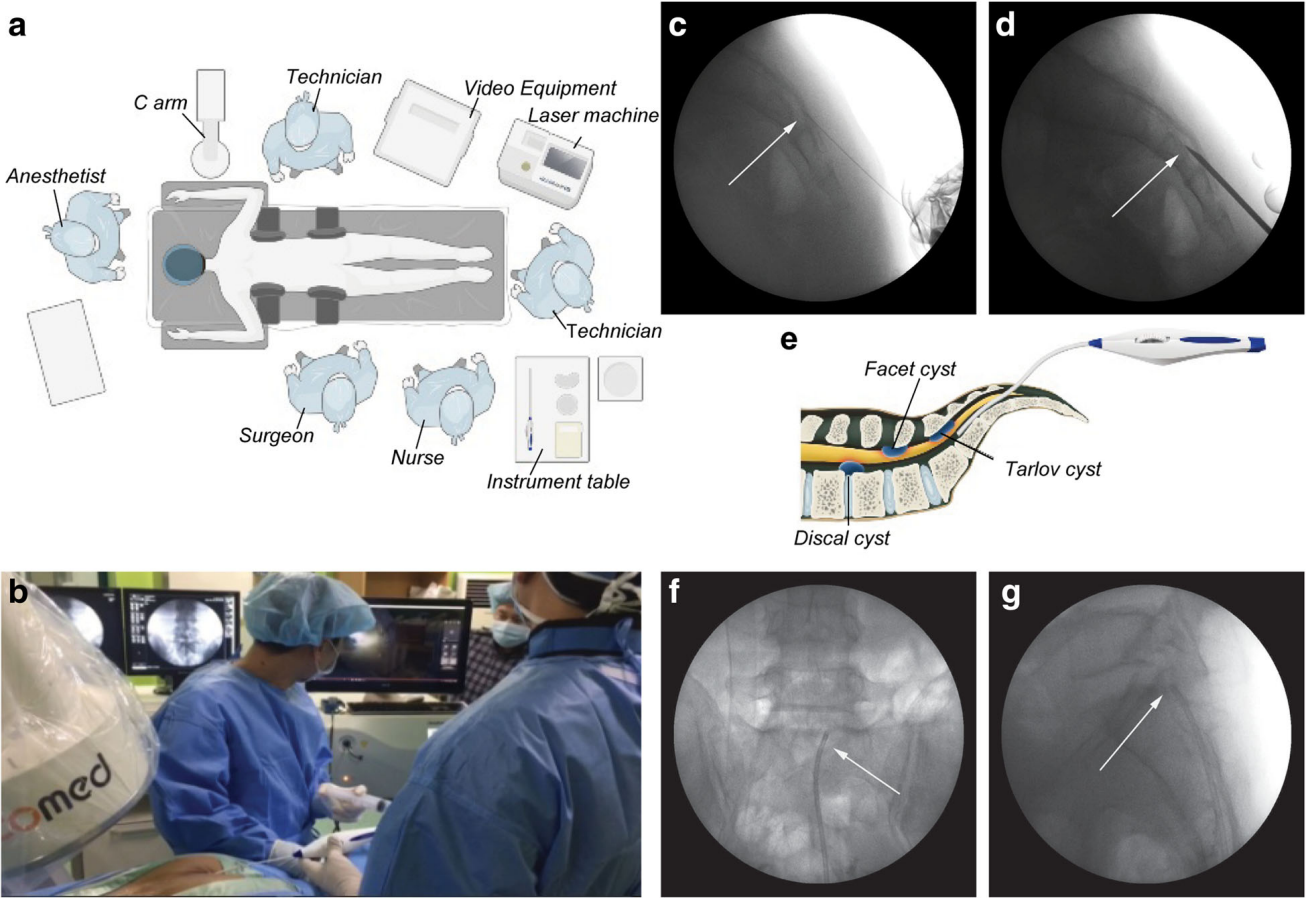
**a** Operating room arrangement. **b** After the epiduroscope is positioned at a suitable site with a C-arm, laser vaporization is performed under high-definition video monitoring. **c** Local anesthesia is applied to the skin and tissues surrounding the sacral hiatus (arrow). **d** A tapered cannula is inserted into the epidural space for epiduroscopy (arrow). **e** Schematic illustration of epiduroscopic ruptures according to the type of cyst. **f** Anterior-posterior X-ray image distinguishes the location of the lesions. **g** The lateral radiograph shows the epiduroscopic location, differentiating between ventral and dorsal positions

All surgical procedures were performed in the prone position on a radiolucent spine table. After aseptic draping in the usual manner, local anesthesia (lidocaine 1%, 10 mL), intravenous analgesia (pethidine, 100 mg/2 mL), and intermittent sedation (midazolam, 5 mg/5 mL) were used for pain control and sedation. The arrangement of the surgical room is depicted in Fig. 1a, and the operative scene in Fig. 1b. The entry point into the skin was generally at the midline of the sacral hiatus, which was confirmed using a C-arm. After infiltrating the skin at the entry point with local anesthetic, a 21-gauge needle was inserted under radiographic guidance (Fig. 1c). Epidurography was performed using iodinated contrast medium to confirm the location of the cyst and surrounding neural structures. Next, a 0.3-mm incision was made in the skin at the entry point, and a tapered cannula was inserted through the sacral hiatus (Fig. 1d). A camera-equipped epiduroscope (iDolphin®, Meta Biomed Co., Ltd., Korea) was then inserted into the target point, and the cyst was visualized.

The principles of the procedures, according to the type of cyst, are illustrated in Fig. 1e. The target point of the epiduroscopic catheter was checked against radiographic information regarding the location of the cyst obtained before surgery and confirmed on MRI using anterior-posterior and lateral views (Fig. 1f, g). For safety, discrimination of neural tissue was performed at low power (0.6 J and 6 Hz). Ablation and fenestration was performed at 1.0 J and 10 Hz. The cysts were ruptured using a Holmium: YAG laser (2100-nm Ho: YAG laser, Holiwon®, Wontech, Korea). After the cystic mass was decompressed, the epiduroscope was removed and a sterile dressing applied. In the cases of discal and facet cysts, epidural adhesiolysis was performed using 10 mL of a mixture of 0.5% bupivacaine and 1500 IU hyaluronidase. Contrast media were administered using an epiduroscopic catheter after the mass was sufficiently decompressed, which indicated favorable outcomes due to patency of nerve decompression and mobility. The small insertion point was treated with a reinforced skin closure (Steri-strip®, 3M Inc., Maplewood, MN, USA) without sutures.

### Outcome measures

Changes in back and leg pain following surgical decompression of cysts using ENLD were evaluated using a 1–10 visual analog scale (VAS). Improvement in function following surgery was evaluated using the Oswentry Disability Index (ODI) [14].

### Statistical analyses

All parameters were analyzed statistically. Data are presented as mean and standard deviation (mean ± SD). Differences between pre- and post-operative VAS pain scores and ODI scores were compared non-parametrically using separate Wilcoxon signed-rank tests. Differences were considered statistically significant if *p* values were less than 0.05.

## Results

All ten patients underwent minimally invasive cyst rupture using ENLD technique with Holmium: YAG laser. The average duration of follow-up for participants in the study was 12.6 ± 1.0 months. The average duration of cyst surgery was 21.3 ± 3.0 min. The average length of participants’ hospital stay was 1.5 ± 0.5 days (Table 1).

**Table 1:** Patient characteristics

Characteristics		Mean (SD)
Age, mean (in years)		46.6 ± 14.2
Sex (%)	Male	6 (60.0)
	Female	4 (40.0)
Type of symptom, *N* (%)	Pain only	5 (50.0)
	Pain and weakness	5 (50.0)
Symptom duration (months)		4.7 ± 0.7
Follow-up duration (months)		12.6 ± 1.0
Operation time (minutes)		21.3 ± 3.0
Hospital stay (day)		1.5 ± 0.5
Type of cyst (based on radiologic findings), *N* (%)	Discal cyst	2 (20.0)
	Facet cyst	4 (40.0)
	Pavlov cyst	4 (40.0)

Analysis of VAS scores revealed significant improvement in both back and leg pain from pre- to post-surgery (Table 2). Mean VAS scores for back pain decreased from 4.7 ± 0.7 to 1.8 ± 0.8 on the last follow-up (*p* < .001). Mean VAS scores for leg pain decreased from 5.8 ± 1.2 to 1.6 ± 0.7 (*p* < .001). Mean ODI scores (%) decreased from 27.2 ± 10.7% to 14.6 ± 7.7% post-surgery (*p* < .001). There were no significant complications, such as post-operative infection or hematoma, or a need for a repeat surgery. During the follow-up period, there was no symptom recurrence that warranted a repeat surgery.

**Table 2:** Clinical outcomes of the study group

	Pre-operative scores	Post-operative scores	*p* value
Visual analog scale back pain	4.7 ± 0.7	1.8 ± 0.8	0.001
Visual analog scale leg pain	5.8 ± 1.2	1.6 ± 0.7	0.001
Oswentry Disability Index (%)	27.2 ± 10.7	14.6 ± 7.7	0.001

Differences between pre- and post-operative visual analog scale pain scores and Oswentry Disability Index scores (%) were compared non-parametrically using Wilcoxon ranked-sum tests (*α* = 0.05)

### Report of the cases

#### Case 1

**Fig. 2:**
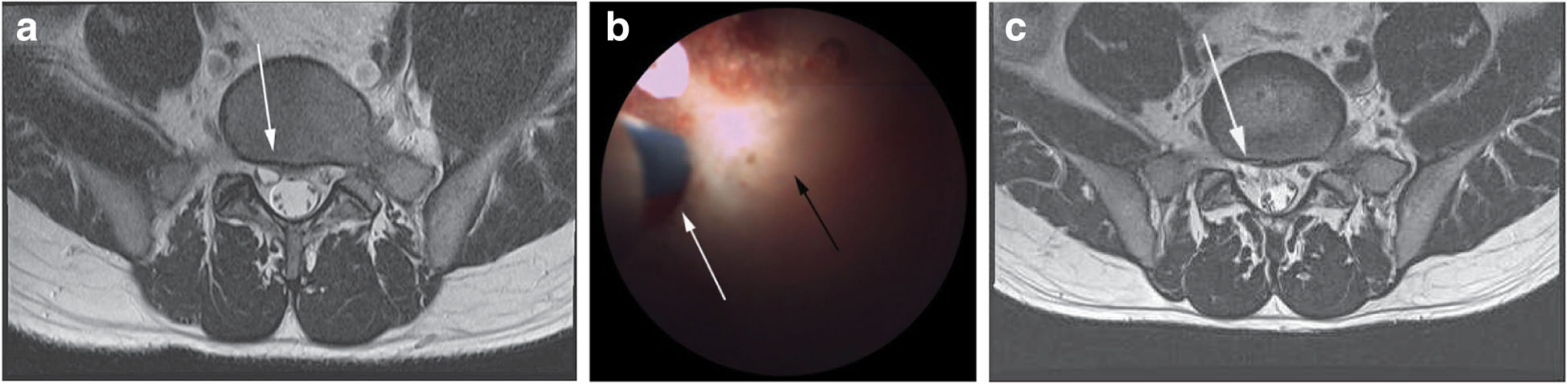
**a** T2-weighted axial magnetic resonance (MR) image shows a cystic mass (arrow) originating from the disc and compressing the traversing right nerve root. **b** Epiduroscopic image shows the laser electrode inserted (white arrow) into the cyst (black arrow). **c** Post-operative T2-weighted axial MR image shows the decompressed cyst (arrow) and visualization of the L5 traversing root

A 26-year-old man visited the hospital on August 21, 2016, with a 6-month history of pain in his right leg. The pain had a radiating quality and had not been relieved by medication. On examination, the patient exhibited monoparesis of the sacral (S) 1-innervated gastrocnemius muscle, with difficulty in pushing the foot, revealing a lower right limb power of 4/5. Other neurological parameters were within normal limits. There was no related familial or past history. Results of MRI performed on August 23 showed a single fluid-containing lesion at the lumbar (L) 4–5 level measuring 8 × 6 mm (Fig. 2a); the nerve root traversing L5 could not be identified due to the mass effect of the lesion. The patient received ENLD on August 24, 2016. After confirming adequate positioning of the epiduroscope, multiple laser fenestrations (1.0 J, 10 Hz) were performed. Endoscopic visualization showed decompression of the cystic mass (Fig. 2b). The patient reported immediate improvement of his radiating pain following the procedure. The MR images acquired 2 weeks post-procedure showed that the lesion had significantly regressed, and post-operative MRI was now able to identify the nerve root traversing L5 (Fig. 2c).

#### Case 2

A 52-year-old woman presented at the hospital on October 21, 2017, with a 12-month history of low back pain and right leg numbness. Her back pain had increased progressively, and she had developed radiating pain in her right leg along with hypoesthesia. Physical examination showed normal motor strength and deep tendon reflexes. There was no related past or familial history. She was initially prescribed non-steroidal anti-inflammatories (NSAIDs) for more than 6 months, but her symptoms did not improve, at which point she was transferred to our clinic.

**Fig. 3:**
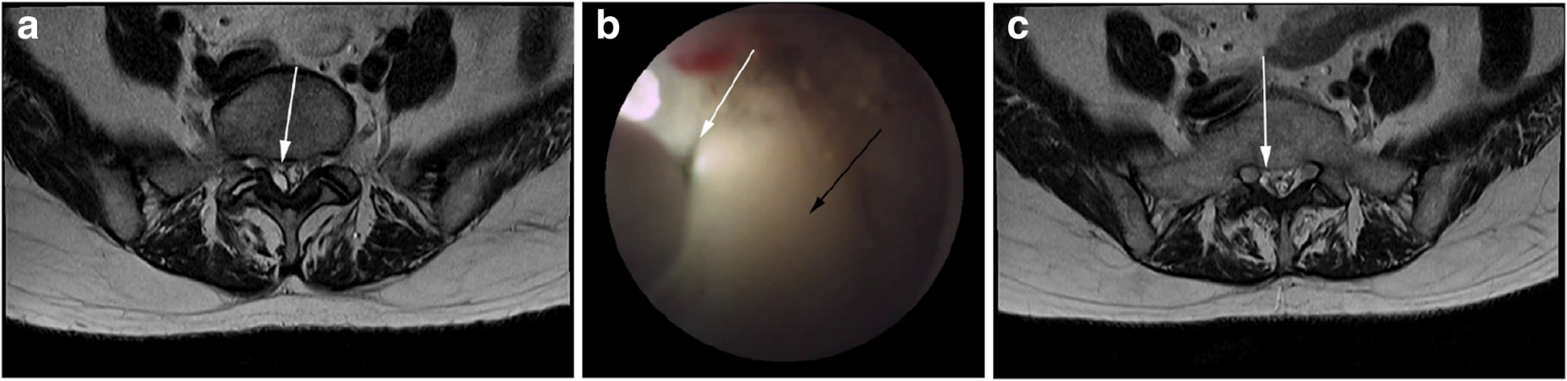
**a** Pre-operative magnetic resonance (MR) image shows a facet-originated cyst (arrow) compressing the thecal sac sacral (S) 1 nerve root. **b** Epiduroscopic image shows the laser electrode inserted into the cystic mass (white arrow); the mass decreased in size (black arrow) post-surgery. **c** Post-operative MR image shows the decompressed cyst (arrow) and the released thecal sac and S1 nerve root

The MR images showed a single fluid-containing lesion at the L5–S level measuring 12 × 8 mm and originating from the right interarticular facet of the lesion (Fig. 3a). The L5 and S1 root were compressed by the cystic mass. After adequate positioning of the epiduroscope, the mass was identified under video monitoring. Stimulation and laser fenestration were performed as described in case 1. Epiduroscopic images showed multiple ruptures in the cyst and cyst decompression (Fig. 3b). Steroid and normal saline irrigation were performed before the incision closure. The patient’s pain and neurological symptoms improved in the first 2 weeks post-procedure. In addition, MR images of the lesion acquired 2 weeks post-procedure showed a deflated cyst measuring 6 × 6 mm (Fig. 3c).

#### Case 3

**Fig. 4:**
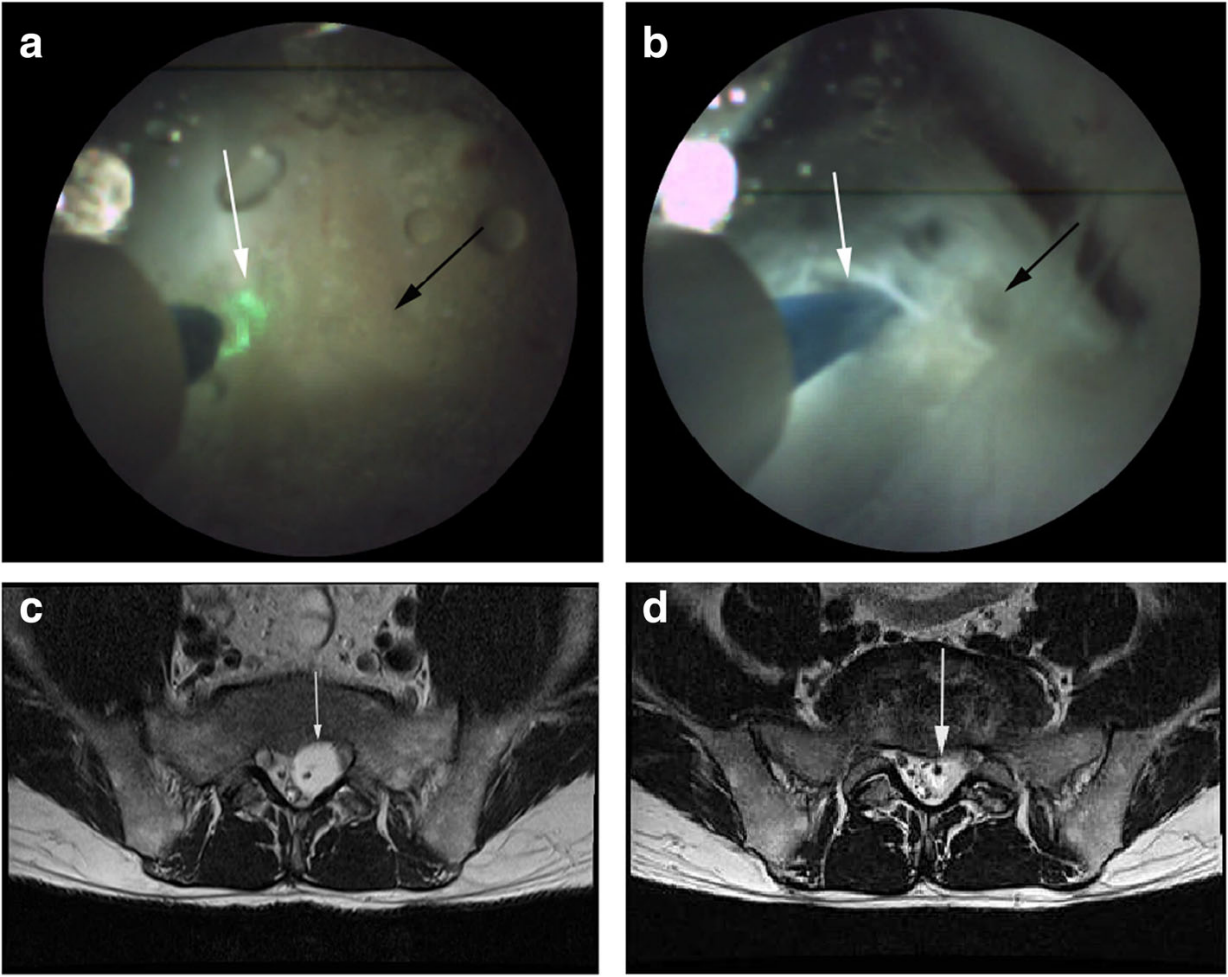
**a** Epiduroscopic image shows a laser electrode inserted into the cystic mass (white arrow) and the confirmed mass (black arrow). **b** Epiduroscopic image shows a laser electrode inserted into the cystic mass (white arrow); after decompression, cerebrospinal fluid was expelled and the cyst decompressed (black arrow). **c** T2-weighted axial magnetic resonance (MR) image shows the inflated cyst containing cerebrospinal fluid and occupying the spinal canal (white arrow). **d** Post-operative MR image shows the released dural sac and deflated Tarlov cyst (white arrow)

A 44-year-old man visited the hospital on November 27, 2016, with a history of buttock and leg pain. The buttock pain had persisted for more than 8 months, while the leg pain had presented 4 weeks ago. Physical examination showed normal motor strength, reflexes, and sensory capability. There was no related past or familial history. He was initially managed with pain medication (NSAIDs) and physiotherapy symptomatically. Subsequent MRI of the spine revealed a large cystic lesion at the S1–2 level with T2 high signal intensity. The size of cystic mass was 50 × 45 mm; due to the mass effect of the cyst, the S1 and S2 nerve roots on the left had been moved to the right side, and the right S1 root could not be identified on imaging. When the epiduroscope was advanced merely 50 mm from the sacral hiatus, we identified a well-defined cystic mass at the S2 level. Cyst rupture was performed at a low voltage (0.6 J, 6 Hz) to protect surrounding neural structures (Fig. 4a). After epiduroscopic confirmation of decompression (Fig. 4b), fluoroscopic imaging also showed iodine filling. The Tarlov cyst that was inflated preoperatively (Fig. 4c) showed decrease in size, and the dural sac was decompressed (Fig. 4d). The patient’s pain and neurological symptoms improved within the first week.

## Discussion

Patients with degenerative changes in the lumbo-sacral region often present with pain and neurological deficits. However, benign cysts, such as discal, juxtafacet, and perineural cysts also commonly present with radiating pain because of their anatomical location, which can interfere with the traversing and exiting nerve roots [15, 16].

The cysts in the first case presented here originated in the intervertebral space. Gas-filled cysts in the intervertebral space which share space with the posterior longitudinal ligaments have been previously described as a cause of radiculopathy [4, 17, 18]. This pathological condition is usually located ventrally. The vacuum phenomenon, which is caused by a reduction of discal pressure due to flexion and traction movements [18], has been suspected to be the cause of gas formation, which in turn compresses the nerve root and thecal sac.

The second case was that of a synovial cyst. It is one of the most common benign extradural masses found in the spine. Synovial cysts within the facet joints have been reported to cause nerve compression [19, 20]. The last case we presented (case 3) involved a perineural (Tarlov) cyst. The etiology of this condition remains unclear. Congenital formation, hemosiderin deposition, breakage of venous drainage, and arachnoidal proliferation around the sacral nerve root are current hypotheses [21, 22].

Although radiologically, benign lumbo-sacral cysts are typically asymptomatic [23], some cases do produce symptoms. The build-up of gas in a discal cyst, overflow of synovial fluid in a facet cyst, and build-up of pulsatile and hydrostatic pressure in a perineural cyst are all considered to be likely causes for lumbo-sacral cyst formation and symptom progression [6, 24]. Space-occupying cysts can cause neurological changes or radicular pain via compression, pulling forces, or distortion [25].

Currently, there is no consensus regarding the best treatment for symptomatic cystic lesions. Surgical procedures have shown favorable outcomes, but neurological deterioration and sequelae of laminectomy have also been reported [7]. Fluoroscopic and CT-guided rupture have an 84% success rate, but 25% of the patients undergo subsequent surgeries due to incomplete cyst decompression during the original procedure [8]. The trans-foraminal approach has been attempted in some cases, but this procedure can only be applied in patients with intact foramen without senile changes.

Epiduroscopic management using the Holmium: YAG laser is an emerging minimally invasive procedure without the risk of bone and ligament injury, compared with other surgical procedures, that may be applied for many spinal conditions. It has undergone trials for the treatment of herniated lumbar discs, spinal stenosis, failed-back-syndrome, and facet cysts [26]; ENLD yields superior results relative to caudal epidural injection [27] and physiotherapy [28]. Since 2007, high-definition (HD) epiduroscopic visualization has been available, allowing better visibility and neural tissue identification compared with the previous version; ENLD has achieved sufficient decompression and fenestration via laser vaporization for various pathological conditions [29].

### Limitations

There are some limitations of this study which must be acknowledged. Firstly, the data in this study was acquired from a small trial with no control group. Therefore, it is possible that the potential benefit of undergoing ENLD for patients was overestimated. It is also possible that patient selection for the study, which included patients with symptomatic cysts, influenced the outcomes. Secondly, remnant cysts can recur due to the characteristics of the fenestration procedure, and the short follow-up duration underestimates this possible complication. Despite these limitations, we believe that this study is clinically important and demonstrates a novel minimally invasive treatment approach for benign cystic mass.

## Conclusions

The results of this study suggest that ENLD with HD visualization and Holmium: YAG laser can be used to treat benign spinal cysts safely, precisely, and effectively; ENLD renders cyst fenestration via epiduroscopy possible without incision and bony destruction. Although this is a preliminary report for a novel technique, ENLD for symptomatic cysts may be a valuable alternative to surgery. Although ENLD was not associated with post-surgical complications for any of the participants, careful follow-up is necessary. Large, randomized, multicenter trials are needed to further explore the potential of ENLD.

## Abbreviations

CSF: Cerebrospinal fluid
CT: Computed tomography
ENLD: Epiduroscopic neural laser decompression
HD: High definition
MRI: Magnetic resonance imaging
NSAID: Non-steroidal anti-inflammatory drugs
ODI: Oswentry Disability Index
SD: Standard deviation
VAS: Visual analog scale
